# Papers Please - Predictive Factors of National and International Attitudes Toward Immunity and Vaccination Passports: Online Representative Surveys

**DOI:** 10.2196/32969

**Published:** 2022-07-15

**Authors:** Paul M Garrett, Joshua P White, Simon Dennis, Stephan Lewandowsky, Cheng-Ta Yang, Yasmina Okan, Andrew Perfors, Daniel R Little, Anastasia Kozyreva, Philipp Lorenz-Spreen, Takashi Kusumi, Yoshihisa Kashima

**Affiliations:** 1 Melbourne School of Psychological Sciences The University of Melbourne Melbourne Australia; 2 Unforgettable Research Services Melbourne Australia; 3 School of Psychological Science The University of Bristol Bristol United Kingdom; 4 School of Psychological Science The University of Western Australia Perth Australia; 5 Department of Psychology National Cheng Kung University Tainan City Taiwan; 6 Graduate Institute of Mind, Brain and Consciousness Taipei Medical University Taipei Taiwan; 7 Centre for Decision Research Leeds University Business School University of Leeds Leeds United Kingdom; 8 Center for Adaptive Rationality Max Planck Institute for Human Development Berlin Germany; 9 Graduate School of Education Kyoto University Kyoto Japan

**Keywords:** COVID-19, immunity passport, vaccination passport, cross-cultural, health policy, digital certificates, SARS-CoV-2, vaccine, policy, international

## Abstract

**Background:**

In response to the COVID-19 pandemic, countries are introducing digital passports that allow citizens to return to normal activities if they were previously infected with (immunity passport) or vaccinated against (vaccination passport) SARS-CoV-2. To be effective, policy decision-makers must know whether these passports will be widely accepted by the public and under what conditions. This study focuses attention on immunity passports, as these may prove useful in countries both with and without an existing COVID-19 vaccination program; however, our general findings also extend to vaccination passports.

**Objective:**

We aimed to assess attitudes toward the introduction of immunity passports in six countries, and determine what social, personal, and contextual factors predicted their support.

**Methods:**

We collected 13,678 participants through online representative sampling across six countries—Australia, Japan, Taiwan, Germany, Spain, and the United Kingdom—during April to May of the 2020 COVID-19 pandemic, and assessed attitudes and support for the introduction of immunity passports.

**Results:**

Immunity passport support was moderate to low, being the highest in Germany (775/1507 participants, 51.43%) and the United Kingdom (759/1484, 51.15%); followed by Taiwan (2841/5989, 47.44%), Australia (963/2086, 46.16%), and Spain (693/1491, 46.48%); and was the lowest in Japan (241/1081, 22.94%). Bayesian generalized linear mixed effects modeling was used to assess predictive factors for immunity passport support across countries. International results showed neoliberal worldviews (odds ratio [OR] 1.17, 95% CI 1.13-1.22), personal concern (OR 1.07, 95% CI 1.00-1.16), perceived virus severity (OR 1.07, 95% CI 1.01-1.14), the fairness of immunity passports (OR 2.51, 95% CI 2.36-2.66), liking immunity passports (OR 2.77, 95% CI 2.61-2.94), and a willingness to become infected to gain an immunity passport (OR 1.6, 95% CI 1.51-1.68) were all predictive factors of immunity passport support. By contrast, gender (woman; OR 0.9, 95% CI 0.82-0.98), immunity passport concern (OR 0.61, 95% CI 0.57-0.65), and risk of harm to society (OR 0.71, 95% CI 0.67-0.76) predicted a decrease in support for immunity passports. Minor differences in predictive factors were found between countries and results were modeled separately to provide national accounts of these data.

**Conclusions:**

Our research suggests that support for immunity passports is predicted by the personal benefits and societal risks they confer. These findings generalized across six countries and may also prove informative for the introduction of vaccination passports, helping policymakers to introduce effective COVID-19 passport policies in these six countries and around the world.

## Introduction

The SARS-CoV-2 virus responsible for COVID-19 has infected more than 360 million individuals worldwide and resulted in more than 5.6 million deaths [1]. As the virus continues to spread, countries seek ways to restart their economies and allow citizens to move freely without reigniting the pandemic. Vaccines are the foremost tool in combating the virus, and countries are introducing “vaccination passports” to allow low-risk individuals to travel, work, and gather under lowered restrictions [2,3]. However, there remains a stark gap between international vaccination programs, with many, predominantly poorer, countries lacking vaccines and still waiting to administer their first dose [4]. Additionally, it is unclear how effective current vaccines will be against newly emerging virus variants [5,6]. In countries where vaccines are limited, or where virus variants outpace vaccine effectiveness, immunity passports may be used.

Immunity passports identify previously infected and now recovered individuals by testing for SARS-CoV-2 antibodies [7]. Like vaccinated individuals, recovered individuals are thought to have a lower likelihood of contracting, spreading, and experiencing the most severe symptoms of the virus [7]. A recent World Health Organization report [8] suggests that recovered individuals develop antibodies within 4 weeks following infection, that immune responses remain robust for 6-8 months, and that, due to the manner by which vaccines target a specific spike protein, naturally acquired antibodies may be more robust to emerging virus variants (vaccines are effective against current variants of concern [eg, Delta and Omicron]). As such, immunity passports may prove useful in the fight against COVID-19, especially when used in conjunction with vaccination passports. Indeed, the European Union [9] has proposed exactly this with their new “green card,” a digital certificate that will act as both a vaccination and immunity passport. For simplicity, we refer to these vaccination and immunity passports collectively as “immunization passports.”

Immunization passports may allow economies to rapidly bounce back, with individuals perceiving crowded shops and workplaces as safer if others are recovered or vaccinated [10]. Similarly, businesses may require proof of immunization to enter their premises or use their services [11], and countries may require proof of immunization to cross their borders [2]. For example, the International Air Transport Association has developed the “Travel Pass” app [12] to store a COVID-19 vaccination record on the user’s phone, such that data can be shared with governments and transport authorities before accessing flights and crossing a country’s border.

Additional privacy measures may accompany these apps, as is the case with South Korea’s “Green Pass” [3], a vaccination certificate that uses blockchain technology to make passes both shareable and tamper-proof [13]. Australia uses an alternative method, issuing international passes as QR codes protected with visible digital seals (nonconstrained) and administered only after one’s data have been verified by the federal Australian Passport Office [14]. These immunity passport apps and QR codes are a technological extension of existing vaccination requirements such as the physical “yellow card” that accompanies yellow fever vaccination, which is necessary to enter many countries in Africa and Central and South America [15].

The potential introduction of immunization passports carries a host of scientific, legal, and ethical questions such as: Are recovered and vaccinated individuals immune to new virus variants [8]? Will these passports become a legal requirement, and how will people who cannot risk becoming infected or cannot get vaccinated be impacted? Will individuals try to become infected if doing so confers additional freedoms? [16] Each of these questions is critical to national health policies and has been a source of recent debates between privacy advocates and politicians in Britain [17], and the cause of public protests in France [18]. World governments and health policy decision-makers need scientifically informed answers to two key questions: Will people around the world accept and support the use of immunization passports? And if so, why?

We narrow the scope of our investigation to the introduction and acceptance of immunity passports—instances where an individual has been infected and recovered—in six countries around the world, as immunity passports may yet prove relevant to countries both with and without vaccination programs. Of course, these findings may also prove insightful and may extend to the conditions necessary for vaccination passport acceptance. Key to the current investigation is understanding what societal, personal, and contextual factors influence immunization passport acceptance.

Societal factors may shape one’s attitude toward whether immunization passports will benefit the community at large, thereby influencing passport acceptance [19]. Health policy acceptance may improve with a sense of communal (rather than individualistic) responsibility for the public’s well-being [20]. Similarly, acceptance may improve or diminish with perceptions of shared societal experiences such as stay-at-home “lockdowns” [21] and the perceived effectiveness of government COVID-19 policies (eg, COVID-19 vaccine uptake improves with perceived government effectiveness and trust in government) [22].

Personal experiences may also affect one’s attitude toward using an immunization passport. For example, having had or known someone who has had COVID-19 may incentivize one toward the use of immunity passports [19]. Strong neoliberal worldviews—a belief that the free market is fair and sensitive to the social and financial needs of the people—and a desire to return to normal economic activities may also affect passport acceptance [23]. Similarly, higher education may prove important to shaping one’s opinions regarding the equality and necessity of immunity passports, just as it has with vaccinations [24].

Finally, immunity passport acceptance may depend on contextual factors regarding the state of the pandemic such as COVID-19 cases, deaths, and vaccine progress, which may change country to country and across time. In developing an understanding of what factors influence immunization passport acceptance, we may consider (1) acceptance while attempting to control for the contextual influences of each country (an international model), and (2) acceptance dependent on each country (national models). The former informs us of the necessary conditions for immunity passport acceptance across countries, allowing our findings to potentially generalize beyond our sample of six countries. By contrast, the latter assesses acceptance within each sampled country and may show how it varies as a function of each country’s individual context and culture.

In summary, the objective of this study was to identify which societal, personal, and contextual factors predict the uptake of immunity passports across six countries. International modeling was used to provide generalizable findings, while national modeling was used to look for factors that deviate from international interpretations. Bayesian statistics were used to provide evidence toward or against factors that predict the uptake of immunity passports. This work is intended to provide clear scientific findings for medical and health researchers and for policymakers.

## Methods

### Design

We surveyed attitudes toward immunity passports in six countries with different experiences during the COVID-19 pandemic: Australia, Germany, the United Kingdom, Spain, Japan, and Taiwan. Using Bayesian linear mixed models, we aimed to determine which factors—societal, personal, and contextual issues related to COVID-19—influenced immunity passport acceptance. We examined our data in two ways. First, we attempted to control for the idiosyncratic effects of each country on immunity passport acceptance (using random effects in our modeling) to create a generalized framework for immunity passport acceptance. Second, we assessed acceptance within each country to consider cultural and contextual differences.

### Ethics Considerations

All participants read a plain-language statement describing the online survey, the research question—to understand what factors contribute to the uptake of COVID-19 tracing technologies and immunity passports—and the study’s benefits, risks, and data protections, before providing informed consent. Participants were informed that data collection would occur through password-protected accounts, be transferred through encrypted networks, and be held on secure password-protected servers; that no identifying information would be published or released; and that anonymized data would be made available through the Open Science Framework (OSF). Australian participants were reimbursed with gift cards or points programs per their agreement with Dynata, Spanish and German participants per their agreement with Lucid, Japanese participants per their agreement with Cross Marketing, and Taiwanese participants per their agreement with Gosurvey. UK participants were reimbursed a flat rate of £0.85 (~US $1.07) per 10-minute survey. Ethics approval was obtained for data collection in Australia and Japan from the University of Melbourne (approval 1955555), in the United Kingdom from the University of Bristol (approval 103344), in Germany from the Max Planck Institute for Human Development (approval L2020-4), in Spain from the University of Leeds (approval 103402), and in Taiwan from the National Cheng Kung University (approval 108-072).

### Participants

Table 1 displays demographic information for each country and sample. We sampled 13,678 participants across six countries to determine their attitudes toward and acceptance of immunity passports. Each country collected between one and four nationally representative online samples. Samples were stratified by age, gender, and, where possible, state or province, based on the country’s most recent census data. Participants were aged 18 years or older, and completed a 10- to 15-minute online survey for which they were financially reimbursed. Representative samples were obtained using third-party recruitment services and assigned unique identifiers upon entering the survey to ensure anonymity. Further country-specific details are provided in Multimedia Appendix 1. Data collection was completed as part of a wider international collaboration examining the acceptability of mobile tracking technologies to address the COVID-19 pandemic [23,25-28]. However, previous publications did not address the uptake of immunity passports.

**Table 1:** Demographic information relevant to each sample within each country.

Characteristic		Australia		Germany	Japan	Spain	Taiwan					United Kingdom
		Sample 1	Sample 2				Sample 1	Sample 2	Sample 3	Sample 4		
Participants, n		1514	578	1514	1081	1505	1500	1500	1500	1500	1486	
Age (years), mean (SD)		48 (17)	48 (17)	47 (16)	46 (17)	48 (16)	40 (12)	40 (12)	40 (12)	41 (12)	46 (16)	
**Gender, %**												
	Man	50	48	49	49	48	48	47	48	50	48	
	Woman	49	51	50	51	52	52	53	52	50	51	
	Other	<1	<1	<1	0	<1	0	< 1	<1	0	<1	
	Prefer not to say	<1	0	0	0	0	0	0	<1	<1	<1	
**Education, %**												
	Less than high school	9	11	14	3	10	1	1	1	1	16	
	High school graduate	37	40	63	39	42	12	14	13	13	17	
	University graduate	54	49	23	58	47	87	86	86	86	67	

### Procedure

As the pandemic evolved, survey designs were updated with each sample; however, the key design elements assessed in this study remained unchanged (see Figure A1 in Multimedia Appendix 1). Survey questions were designed to address primary factors of the health belief model: illness severity (harm) and sensitivity (concern), policy benefits and barriers, self-efficacy, and calls to action [29,30].

Each participant provided informed consent and demographic information before using a Likert scale to report on their perceptions and impact of the COVID-19 pandemic. Participants then read one of three hypothetical scenarios describing a different type of mobile phone COVID-19 contact-tracing system—telecommunication tracking, a government app, or the Apple/Google exposure notification system—that would alert the user if they had contact with an infected individual, before completing a comprehension check and answering questions about these scenarios (for methods and results on these items by country, see [23,25-27]; note that these studies do not model immunity passport items). Finally, participants read a description of immunity passports before responding to items examining their attitudes toward immunity passports and their neoliberal worldviews. The survey concluded with a study debrief statement (Table 2).

Before responding to the immunity passport items, each participant read the following description:

An ‘immunity passport’ indicates that you have had a disease [or vaccination] and that you have the antibodies for the virus causing that disease. Having the antibodies implies that you are now immune and therefore unable to spread the virus to other people. Thus, if an antibody test indicates that you have had the disease, you could be allocated an ‘immunity passport’ which would subsequently allow you to move around freely. Immunity passports have been proposed as a potential step towards lifting movement restrictions during the COVID-19 pandemic.

Upon survey completion, data were augmented with country-specific information. Data included national indices such as the World Bank’s Perceived Government Effectiveness Scale (scale 0-100, with higher values indicating greater effectiveness) [31], and the individuality subscale from the Hofstede Index of Collectivism (scale 0-100, with higher values indicating a more individualistic, less collectivist culture) [32]. Data also included COVID-19 cumulative cases and deaths [1], mask usage (binary variable: true or false) [33], stay-at-home “lockdown” usage (binary: true or false) [33], and mobile tracking technology usage (eg, COVIDSafe in Australia or the CORONA-WARN-App in Germany) [23,25-28,33]. News articles used to determine national policy metrics (eg, mask usage and lockdowns) are available through the OSF [33].

**Table 2:** COVID-19 perceived risk and impact, immunity passport, and worldview items. IP: immunity passport; WV: world view.

Item	Question	Label
Perception 1	How severe do you think the novel coronavirus (COVID-19) will be for the general population?	General harm
Perception 2	How harmful would it be for your health if you were to become infected with COVID-19?	Personal harm
Perception 3	How concerned are you that you might become infected with COVID-19?	Concern self
Perception 4	How concerned are you that somebody you know might become infected with COVID-19?	Concern others
Impact 1	Have you ever tested positive for COVID-19?	Positive self
Impact 2	Has somebody you know ever tested positive for COVID-19?	Positive other
Impact 3	Have you temporarily or permanently lost your job as a consequence of the COVID-19 pandemic?	Job loss
Passport 1	Would you support a government proposal to introduce “immunity passports” for the novel coronavirus (COVID-19)?	IP Support 1st
Passport 2	How concerned are you about the idea of introducing an “immunity passport” for COVID-19?	IP Concern
Passport 3	How much would you like to be allocated an “immunity passport” for COVID-19?	IP Like
Passport 4	To what extent do you believe an “immunity passport” for COVID-19 could harm the social fabric of your country?	IP Harm
Passport 5	To what extent do you believe that it is fair for people with “immunity passports” to return to work, while those without a passport cannot?	IP Fair
Passport 6	To what extent would you consider purposefully infecting yourself with COVID-19 to get an “immunity passport”?	IP Self-infect
Passport 7	Would you support a government proposal to introduce “immunity passports” for COVID-19?	IP Support 2nd
Worldview 1	An economic system based on free markets unrestrained by government interference automatically works best to meet human needs	WV Economy
Worldview 2	The free-market system may be efficient for resource allocation, but it is limited in its capacity to promote social justice [reverse-scored item]	WV Freemarket
Worldview 3	The government should interfere with the lives of its citizens as little as possible	WV Small Gov

### Data Analysis and Reporting

#### Overview

Anonymized data and analysis codes for this study are available through the OSF [33]. Participants were excluded from analyses for missing a response to the immunity passport support item or for not completing the survey (removed n=790; details in Multimedia Appendix 1). The reported analyses are based on Bayesian methods and credible intervals to determine effects in the data. Bayesian methods sample a posterior distribution of plausible values (the probability that, given our data, the true population mean is “x”) by weighing the likelihood of a given observation against its prior probability of occurring in the sample. Under parametric assumptions, posterior distributions act to constrain the effect of outliers in the tails of the sampled data, allowing the highest region of data density—credible regions of the data distribution—to inform our decisions. Practically, this means that instead of testing a threshold of significance (ie, *P* value or Bayes factor), we may instead compare the 95% credible regions of the data distributions and determine whether or not they overlap.

#### Immunity Passport Perceptions

Bayesian ordinal probit regressions were used to compare Likert-scale responses using the *MCMCoprobit* and *HPDinterval* functions in the R packages *MCMCpack* [34] and *Coda* [35], respectively. This method compares Likert items by assuming there are latent normally distributed continuous variables underlying the ordinal responses. These latent variables are then segmented into ordinal Likert responses by the number of response options minus one as thresholds. To set the location of the underlying latent variable, the lowest threshold parameter is fixed at zero [36] and all other thresholds are estimated. Country-level data were modeled together [37] and individual samples within countries were not modeled. This approach allowed us to directly compare attitudes to immunity passport items across countries and poses more reasonable assumptions than directly comparing the mean or raw distribution of the Likert scales [37]. This analysis was completed for immunity passport items, as presented in the main text, and for COVID-19 perception and worldview items (Multimedia Appendix 2).

#### International Modeling

Bayesian generalized linear mixed effects modeling was used to assess what factors did or did not predict support for immunity passports. Demographics, perceptions, and impact of COVID-19; COVID-19 cases and deaths by country; neoliberal worldviews; and immunity passport items were treated as additive and independent predictor variables of immunity passport support. Random intercept effects were included to account for dependencies introduced in the data by each country. Likert ratings were treated as numeric data and noncategorical variables were scaled within each country to have a mean of 0 and SD of 1.

Posterior distributions of model parameters were estimated using Hamiltonian Markov Chain Monte Carlo No-U-turn Sampling implemented in Stan via the R package *brms* [38,39]. Four chains each with 2000 iterations and 1000 burn-ins were used. Noninformative priors were set for the intercept and random effect SD parameters (both Cauchy distributions centered on 0 and a scale parameter of 2.5), and fixed effects were estimated from weakly informative priors with a Laplacian distribution centered on 0 and a scale parameter of 1. Practically, this means that factors able to overcome this strong prior bias toward zero (ie, no effect) are meaningful.

Models reported in the main text assess passport support after answering immunity passport questions. Outcome variables were reduced to a binary response set: “support yes” (“moderate,” “a lot,” or “extreme” Likert-scale items) and “support no” (“none,” “a bit,” or “some”). Sample order (present in only two counties) and gender “Other” or “Prefer not to say” were removed as small samples led to unstable model fits. The remaining factors had adequate responses for stable model fits. Models predicting passport support prior to answering questions about immunity passports, and models of international attitudes using the full range of ordinal response options are included in Multimedia Appendix 2. All relevant results were comparable to the model presented in the main text.

#### National Modeling

National modeling replicated the model procedures described above; however, only factors within each country were assessed. This allows for cultural and contextual variation to be observed at a national level, which may be informative for readers, researchers, and policymakers in those countries. Modeling for each country is reported separately in Multimedia Appendix 3, and a summary of the primary differences to the international model is presented in the text.

## Results

### Immunity Passport Perceptions

**Figure 1:**
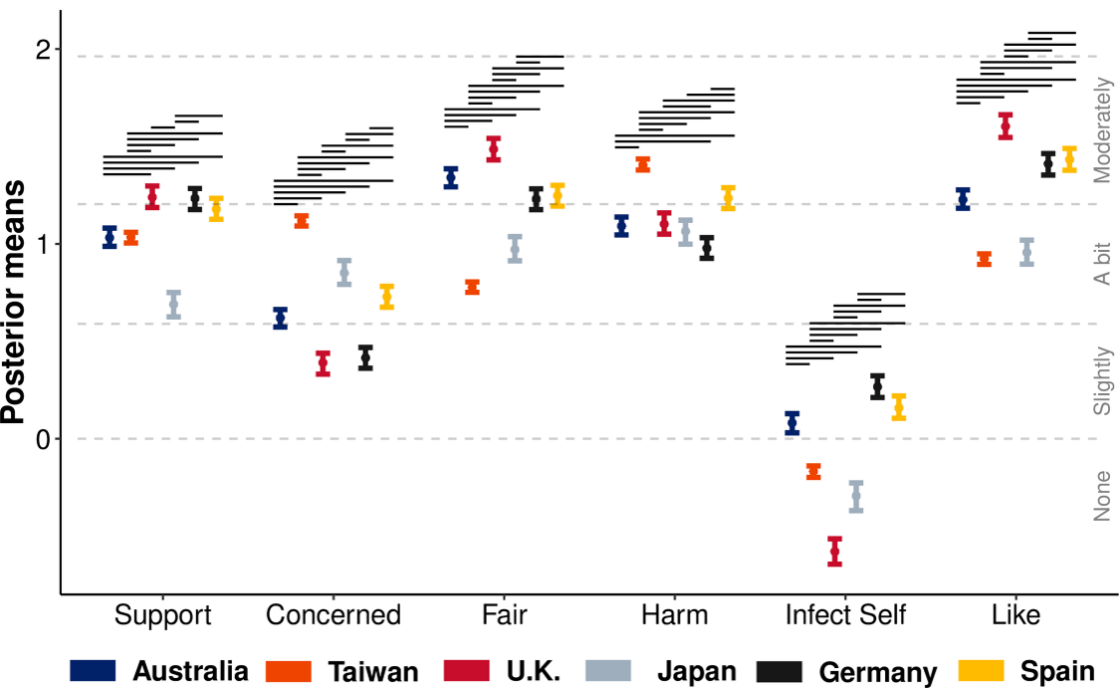
Ordinal regression mean posterior distributions (left axis; vertical error bars) and latent Likert-scale ratings (right axis; dotted horizontal lines) for immunity passport perceptions in Australia, Taiwan, the United Kingdom, Japan, Germany, and Spain. Error bars display the 95% highest posterior density interval. Dotted lines indicate Likert-scale categories, and nonoverlapping intervals (ie, effects) between countries are denoted by black horizontal lines within each item.

Figure 1 displays the mean ordinal regression posterior distributions and associated Likert-style responses for immunity passport perceptions across the six countries. Mean immunity passport support scores based on binary classifications (support: yes=[“moderate,” “a lot,” or “extreme”], no=[“none,” “a bit,” or “some”]) showed that support was the highest in Germany (775/1507, 51.43%) and the United Kingdom (759/1484, 51.15%); followed by Taiwan (2841/5989, 47.44%), Australia (963/2086, 46.16%), and Spain (693/1491, 46.79%); and the lowest in Japan (241/1081, 22.29%). All countries display little to no inclination for infecting one’s self to gain an immunity passport, and although most countries are only “a bit” concerned by the introduction of immunity passports, they are generally deemed as posing a moderate risk of harm to society. As these were secondary analyses to the main focus of this paper, we include a full description of the COVID-19 impact variables and worldview items in Multimedia Appendix 3.

### International Modeling

**Figure 2:**
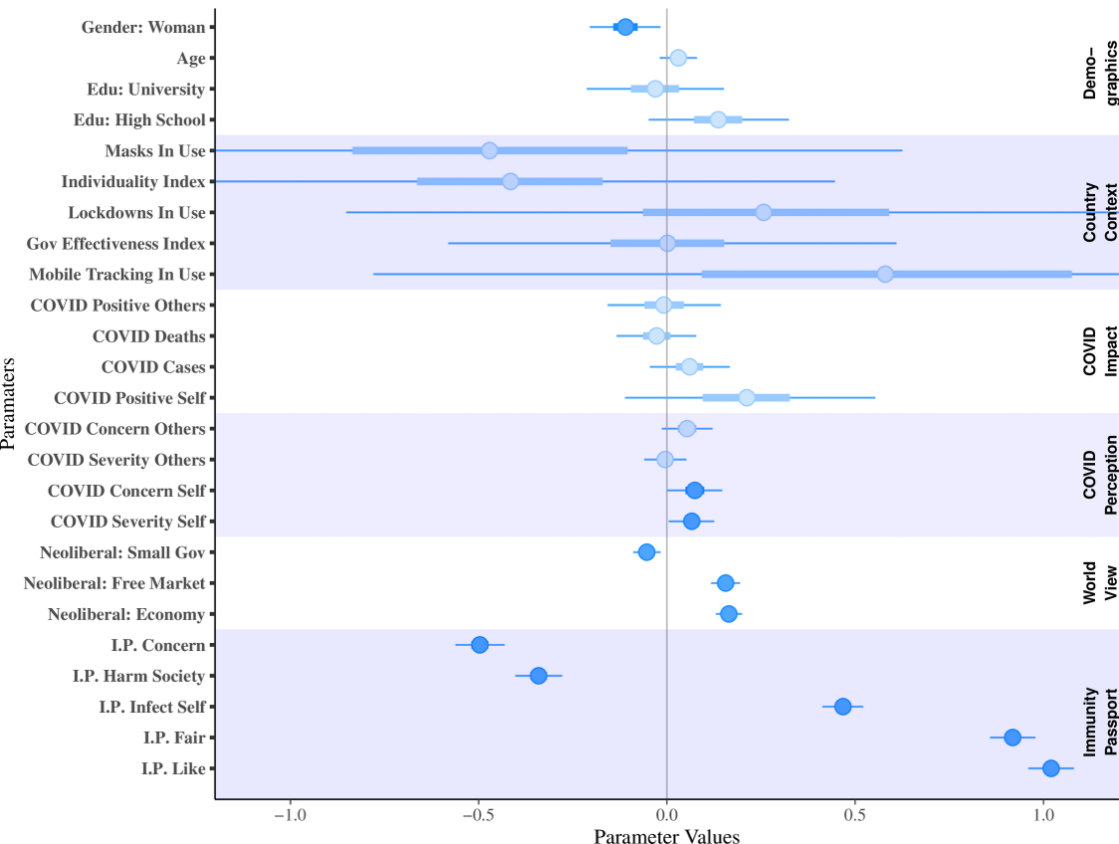
Bayesian generalized linear mixed effects model of immunity passport support (post immunity passport questions) across countries. Positive parameters display immunity passport support; negative values display a decrease in support. Bars represent 50% of the parameter distribution centered on the parameter mean and tails display the 95% highest density interval. Opaque variables show instances where the posterior interval does not overlap zero. IP: immunity passport.

Figure 2 displays the posterior estimates of the Bayesian generalized linear mixed effects model of immunity passport support using demographics, COVID-19 perceptions and impact, country-specific indices (eg, mask usage, government effectiveness), worldview, and attitudes to immunity passports as additive factors, with a random intercept for each country. Error bars display the 95% highest density interval. The global intercept had a mean of –1.67 (95% CI –3.14 to 0.32). Country intercept means were ordered from the lowest to the highest as Japan, Spain, Australia, United Kingdom, Germany, and Taiwan; credible intervals were the lowest for Japan (mean –0.66, 95% CI –2.10 to 0.57) and the highest for Taiwan (mean 0.61, 95% CI –0.79 to 2.31); and intervals for all countries extended over the zero midpoints, indicating no effect. As posterior mean estimates are rather opaque, we provide an explanation of the international model variables in terms of their odds ratios.

Predictive variables of immunity passport acceptance—those where the 95% highest density interval did not cross zero—included increased COVID-19 concern, perceived virus severity to one’s self, worldview (believing the free market works best and that it is limited in its ability to support social justice), and immunity passport items (liking and thinking immunity passports are fair, and being willing to self-infect to receive an immunity passport). Personally liking the idea of immunity passports was the strongest predictor variable, with an odds ratio of 2.8; that is, a 1-SD increase in “liking” immunity passports corresponded to a 2.8-factor SD increase in the odds of supporting their introduction. This may seem rather tautological, but shows that positive attitudes toward immunity passports are the strongest predictor of their acceptance.

Predictive variables against the introduction of immunity passports included gender (identifying as a woman), worldview (supporting minimal government interference), and immunity passport risk items (concern and risk of harm to society). Immunity passport concern was the most predictive item against the acceptance of immunity passports, with a 1-SD increase therein corresponding to a 0.61-factor increase in the odds of supporting the introduction of immunity passports (equivalent to a 1.65-factor increase in the odds of not supporting the introduction of immunity passports).

### National Modeling

Table 3 summarizes the factors that met our criteria for an effect—credible intervals that did not overlap zero—in the international and national models. Parameters are displayed as odds ratios—the degree to which each parameter increases the odds of immunity passport support—and indicate whether they increase or decrease the likelihood of immunity passport support. An odds ratio of 1 indicates no effect, less than 1 indicates a negative relationship, and an odds ratio greater than 1 indicates a positive relationship between parameters. Three notable differences were observed between national and international parameters: gender and COVID-19 severity-self were only identified in the international model, and COVID-19 concern for others was only identified in the national model for Japan.

**Table 3:** Odds ratios for international and national model parameters that did/did not support immunity passport acceptance.^a^

Model	Gender	COVID-19 perceptions			Worldview items			Immunity passport items				
	Woman^b^	Concern self^c^	Severity self^c^	Concern others^c^	Small government^b^	Free market^c^	Economy^c^	Concern^b^	Harm^b^	Infect self^c^	Fair^c^	Like^c^
International model	0.9	1.07	1.07	—^d^	0.98	1.17	1.17	0.61	0.71	1.6	2.51	2.77
Australia	—	—	—	—	0.88	—	1.31	0.64	—	2.03	3.42	3.71
Germany	—	—	—	—	0.9	1.14	1.23>	0.76	0.73	1.58	2.36	3.67
Japan	—	—	—	1.48	—	1.25	—	0.63	—	2.01	1.82	2.56
Spain	—	—	—	—	0.85	1.28	1.12	0.54	—	1.9	3.19	3.29
Taiwan	—	1.14^c^	—	—	—	1.16	1.17	0.61	0.63	1.38	2.27	2.05
United Kingdom	—	—	—	—	—	—	—	0.57	0.53	—	3.16	5.47

^a^Ratios represent the multiplicative increase each coefficient confers to immunity passport support. Displayed parameters are those with credible intervals that did not cross zero.

^b^Column variables that decreases the likelihood of immunity passport support.

^c^Column variables that increases the likelihood of immunity passport support.

^d^Not applicable.

## Discussion

### Principal Findings

The introduction of immunity passports received moderate support across the six sampled countries, except for Japan. International modeling showed that immunity passport acceptance was primarily driven by perceived personal risks (COVID-19 concern and severity, willingness to self-infect) and benefits (liking immunity passports and believing they are fair), and societal factors (neoliberal worldviews). Acceptance was not influenced by contextual factors such as COVID-19 cases and fatalities, or mask, lockdown, or tracing technology usage. National modeling displayed little variation from international results, suggesting that our international findings may prove inferential to the global community.

### International Modeling

International modeling identified several predictive factors of passport support, including worldview, COVID-19 concern for one’s self, and perceived virus severity to one’s self; however, critical variables were those directly assessing attitudes toward immunity passports. Desiring a passport, perceiving passports as fair, and being willing to infect one’s self to gain an immunity passport were all positively associated with immunity passport support. Although immunity passport perceptions displayed greater concern for others than for one’s self, concern and perceived virus severity toward others were not predictive of passport support. These findings highlight that immunity passport support hinges upon personal benefits. Similar findings have been observed for vaccine uptake [40] and for mobile health technologies that emphasize patient self-efficacy [41].

Immunity passport support also improved with neoliberal worldviews, specifically seeing the free market as fair and as working best if unrestrained by government interference. By contrast, limiting government interference was negatively predictive of immunity passport support, along with gender, immunity passport concern, and perceived risk of harm to society. These parameters code societal factors that influence one’s judgment on immunity passport acceptance. Additionally, we posit that these worldview items may serve as a proxy for correlated attitudes such as political worldviews. This may prove important in countries prone to political tribalism (eg, the United States) [42] where bipartisan support would be needed when promoting immunity passports, not from a legislative standpoint but rather from the view of gaining public support and the “social licence to operate” [43,44].

Contextual factors such as COVID-19 policy decisions (ie, wearing masks, home lockdowns, and the introduction of mobile tracking technologies) and country-specific indices (ie, COVID-19 cases and deaths, individualism, government effectiveness) were not predictive of immunity passport support. This reinforces our theory that attitudes toward the uptake of immunity passports are driven primarily by personal risks and benefits, and to a lesser extent, societal factors.

### National Modeling

National modeling revealed minor differences to the international model. Some countries emphasized concern for others (eg, Japan) or concern for one’s self (eg, Taiwan), or differed by their lack of a predictive variable when compared to the international model. For example, immunity passport support increased with the likelihood of infecting one’s self in every country except the United Kingdom. By contrast, some factors were consistently predictive across countries (eg, seeing immunity passports as being “fair”). Understanding international and national variance is key to this study; no single country stands as a monolith from which understanding or predictions may be extrapolated. Attitudes modeled across countries provide insights (eg, the predictive qualities of gender and the severity of COVID-19 to one’s self) otherwise lost at national levels. By contrast, national accounts provide a nuanced view of attitudes that allow policymakers to consider how immunity passports would be perceived within a single country relative to the global community.

### Limitations

The current investigation was primarily limited by our sampling options. Representative online sampling was performed in all countries; however, being online samples, they may be biased toward technological solutions for large-scale problems. Further, samples were not representative for education, with respondents in each country skewing toward being more educated than their respective populations.

We were also severely limited by public perceptions at the time of this investigation. In April-May of 2020, international vaccine rollouts were yet to begin and the focus was on nonpharmaceutical methods for virus suppression. Attitudes may have since shifted as media begin to report on governments seeking to introduce vaccination and/or immunity passports, and the risks and benefits these documents provide. This discussion will only become more heated as corporations such as airlines begin limiting services based on whether individuals have been vaccinated or have recently recovered, and as the long-term side effects of COVID-19 become apparent. Discussions will also evolve as counties reconsider what being “fully vaccinated” entails (eg, one, two, or several booster shots) and what vaccines are deemed suitable for entry to a country. Additionally, public discourse will evolve as people experience the usability of immunization passport technology, as a key barrier for mobile health technology uptake [45].

Finally, a key limitation of our study was our inability to directly assess attitudes to specific security and privacy-preserving digital passport techniques such as blockchain technologies employed by South Korea [3,13] or the Visual Digital Seals (QR code) technology employed by Australia [14]. This omission was due to the survey being conducted before these technologies were in use. Regardless, concern over immunity passports remained a key factor in our modeling, and may be inclusive of privacy and security issues as these are established barriers for the adoption of other nonpharmaceutical COVID-19 interventions (eg, COVID-19 contact-tracing apps) [25,46,47]. Decision-makers should address and minimize these concerns among potential users.

### Conclusion

Governments and corporations are now introducing immunity and vaccination passports to quickly return society and the economy to normal, while encouraging the public to get vaccinated to protect themselves and their loved ones. However, the introduction of these passports will only work if the public supports their use. Policymakers can take from our findings several clear conclusions on how to effectively introduce immunization passports. Passport acceptance will benefit from highlighting the societal benefits (shorter lockdowns, a return to normal work and activities, improved community health) and personal health benefits conferred by these passports, and by addressing and minimizing the societal risks (eg, creating “vaccinated” vs “unvaccinated” social classes) and personal risks (privacy and anonymity) posed by their introduction. To a lesser extent, acceptance would also benefit by framing immunization passports as benefiting the economy and workforce (ie, neoliberal worldview). Finally, we note that internationally, women were less accepting of immunization passports; however, this trend was not observed within individual countries. Hopefully, by successfully accounting for these factors in policy decisions regarding immunity passports, governments and businesses may avoid public backlash when members of the public are prompted: “Papers please?”

## Appendix

Multimedia Appendix 1 Methodological differences between countries and where to find additional methodology details, code, and data for each country.

Multimedia Appendix 2 Alternative ordinal and binomial modeling accounts of the immunity passport data.

Multimedia Appendix 3 Immunity passport modeling completed for each country separately.

## Abbreviations

OSF: Open Science Framework
