# Supplementary material for: Papers Please - Predictive Factors of National and International Attitudes Toward Immunity and Vaccination Passports: Online Representative Surveys
Source: JMIR Public Health Surveill. 2022 Jul 15;8(7):e32969. doi: 10.2196/32969 (PMC9290331; doi:10.2196/32969)
Supplement: Multimedia Appendix 1 [file publichealth_v8i7e32969_app1.docx]

# Multimedia Appendix 1

## Survey differences by country

Due to the rapidly changing nature of the COVID-19 pandemic, surveys were updated regularly and modified for each country. To maximise survey similarities, survey translations were handled by researchers in each of their respective countries (e.g., English surveys were translated to Japanese, Spanish, German, and Chinese), and the survey order was kept the same (Figure A1). The following paragraph details consistent elements across all surveys, based on the Checklist for Reporting Results of Internet E-Surveys (CHERRIES).

All surveys were conducted with internal ethics approval (detailed below). Personal data, such as age, gender, COVID-19 status, were collected in a fully anonymized format using unique identifiers assigned at the start of the survey. Participant provided informed consent after being told the survey would take 10-15minutes (depending on the country and data collection wave), contact details for the responsible researchers, and the purpose of the study – to understand what factors contribute to the uptake of COVID-19 tracing technologies and immunity passports. All surveys were designed and responded to via the Qualtrics survey platform. Participants were contacted through online intermediary recruitment services (e.g., Prolific Academic, GoSurvey), volunteered to be part of the study, and were reimbursed according to their agreement with the recruitment service (detailed below). To prevent bias, items were randomized within question blocks (e.g., a block would represent all items on immunity passports, whereas a COVID-19 impact was assessed as a separate block of items). These details can be observed in the surveys available in both QSF and Word Doc format from our OSF page. Items were displayed one-at-a-time, and completeness checks were conducted via the Qualtrics platform. Participants could go back through the survey to change their answers if necessary. Unique participants were ensured using the closed registration systems offered via the third-party recruitment sites (e.g., Prolific Academic, Dynata, GoSurvey). Incomplete questionnaires were not included in the final sample. Sample weighting was not conducted. In the following section, we briefly highlight primary survey differences between countries, and point to published methods where possible.

### Australia

The Australian surveys were collected on April 15th and May 7th of 2020. The first survey assessed attitudes towards three hypothetical tracing technologies (telecommunication tracing, a Bluetooth Government App, and an App using the Apple/Google Exposure Notification system) and immunity passports. The second survey assessed attitudes towards the Australian COVIDSafe App and attitudes towards immunity passports. A representative sample of the Australian public stratified by age, gender and state were collected through the data sampling platform Dynata, and participants were reimbursed in the form of gift cards, points programs, or charitable contributions as per their agreement with Dynata. The complete surveys may be found via the Australian paper’s Open Science Foundation (OSF) page, https://osf.io/sw7rq/, and the methods in full are reported in Garrett, White, et al. (2021). This study received ethics approval from the University of Melbourne’s psychology health and applied sciences human ethics sub-committee, approval number 1955555.

### United Kingdom

The United Kingdom survey was collected on April 16th 2020 and assessed attitudes towards three hypothetical tracing technologies and immunity passports. A representative sample of 752 participants stratified by age and gender were collected through Prolific Academic, and were reimbursed 85 Pence for completing a 10-minute survey. The survey materials are freely available via OSF, https://osf.io/pw5yj/, and the methods are reported in full in Lewandowsky et al. (2021). This survey did not differ in any meaningful way from the first Australian survey, except in how country specific words were replaced - United Kingdom rather than Australia - for this new audience. This study received ethics approval from the University of Bristol, approval number 103344.

### Germany

The German survey was collected between the 17th – 22nd April and assessed attitudes towards three hypothetical tracing technologies and immunity passports. A representative sample of 1665 participants were collected through the online platform, Lucid, stratified by age, gender and region, and participants were reimbursed per their agreement with Lucid. The full methods for this paper are reported in Kozyreva et al. (2021), and the original surveys may be found in full through the study’s OSF page, https://osf.io/xvzph. This survey did not differ meaningfully from the first Australian survey. The Institutional Review Board of the Max Planck Institute for Human Development approved the surveys (approval L2020-4).

### Spain

The Spanish survey was collected between April 27th and May 5th 2020, and assessed attitudes towards one-of-three hypothetical tracing apps and immunity passport items. As in Germany, a representative sample of 1505 participants were collected through the online data sampling platform, Lucid, and participants were reimbursed per their agreement with Lucid. The Spanish survey was a translation of the United Kingdom survey (reported above), copies of which — along with the associated data — can be access through the Spanish OSF page, https://osf.io/xa4sf. A detailed breakdown of the results from this Spanish sample can be found at https://stephanlewandowsky.github.io/UKsocialLicence/SpainCov1.html. Ethics was obtained for this study through the University of Leeds, ethical approval code: 103402.

### Taiwan

The Taiwan surveys were collected in four waves separated by one-week intervals, starting April 8th 2020 and ending April 29th 2020. Each wave collected a representative sample of 1500 participants (6000 in total) stratified by age, gender, and region, through the survey distribution company, Gosurvey, at a cost of $21,500 USD. Participants were reimbursed per their agreement with Gosurvey. Each survey assessed participant’s psychological resilience, before asking participant’s about their attitudes towards one-of-three hypothetical tracing technologies, and then their attitudes towards immunity passports. The full methods for this paper are reported in Garrett et al. (n.d.), and the surveys and data can be downloaded from the study’s OSF page, https://osf.io/u28n7. These surveys did not differ meaningfully from the first Australian survey. Ethics approval for this study was obtained from the Ethics Committee of the Department of Psychology at the National Cheng Kung University (ethics code 108-072).

### Japan

The Japan survey was collected between May 13th – 14th 2020. A representative sample of 1000 members of the Japanese public were collected through the data sampling company, ‘Cross Marketing’ (‘Kurosu Marketing’), in Japan, and participants were reimbursed based on their personal agreement with Cross Marketing. Participants were queried on their attitudes to one-of-two tracing technologies - a Government Bluetooth App and the Apple/Google exposure notification system - before being assessed on their attitudes towards immunity passports.

Finally, Figure A1 presents the trial structure with participant numbers and exclusions following the Consort transparent reporting of trials structure.


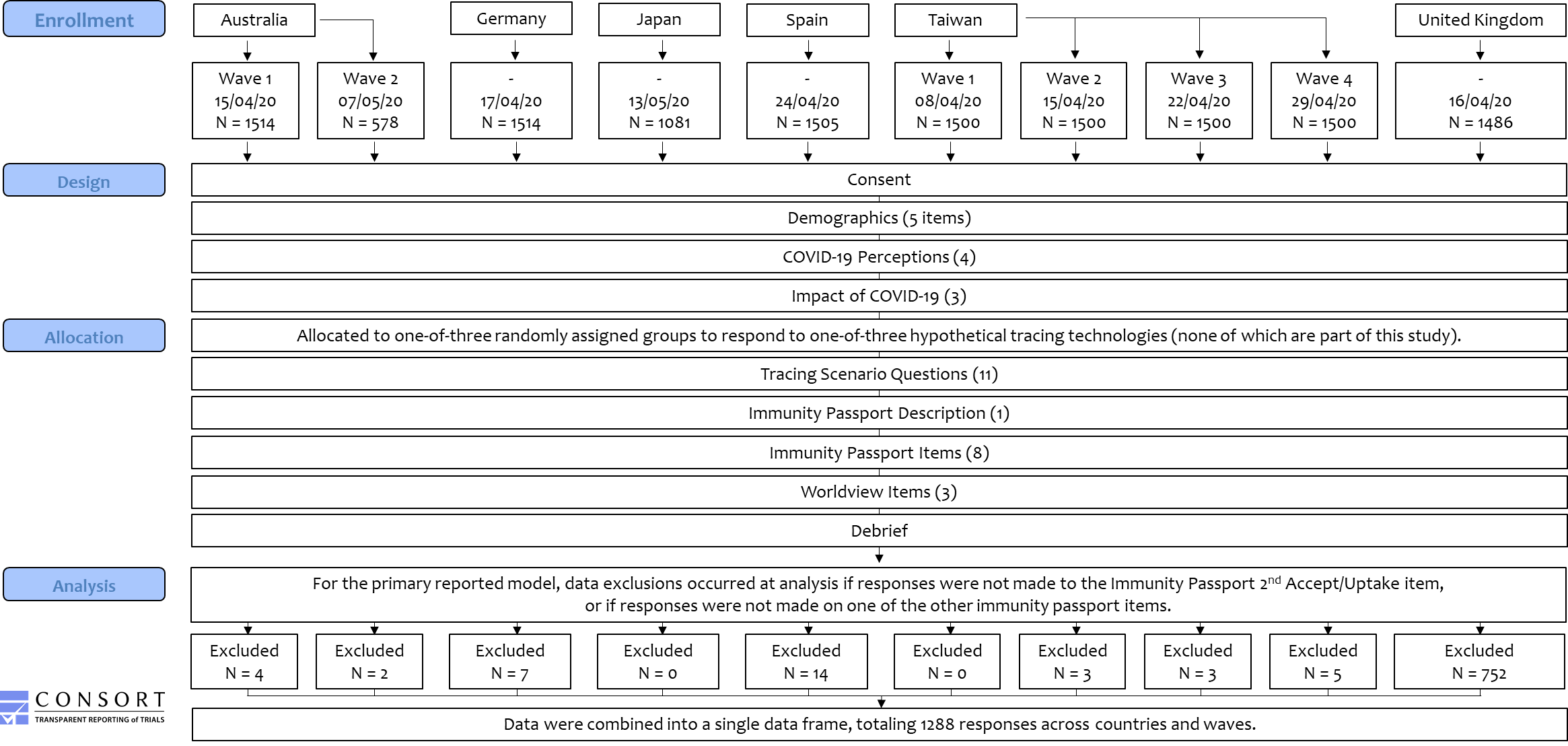


*Figure A1*. Data collection waves by country and date, trial structure, and data analysis exclusions.
