# Supplementary material for: Papers Please - Predictive Factors of National and International Attitudes Toward Immunity and Vaccination Passports: Online Representative Surveys
Source: JMIR Public Health Surveill. 2022 Jul 15;8(7):e32969. doi: 10.2196/32969 (PMC9290331; doi:10.2196/32969)
Supplement: Multimedia Appendix 2 [file publichealth_v8i7e32969_app2.docx]

## Multimedia Appendix 2: Additional Modelling

## COVID-19 Perceptions

Figure B1 (top) shows COVID-19 concerns and perceived severity varied with country, being generally least concerning and severe in Australia and Germany, and most concerning and severe in Spain, the United Kingdom and Japan (see Figure 1 for COVID-19 case numbers). A clear trend within countries shows people are more concerned and view the virus as more severe for others than for themselves.

Figure B1 (bottom) shows attitudes to worldview items were comparable across countries. Overall, attitudes were ‘neutral’ towards a capitalist economy being best, and all countries ‘somewhat agreed’ with the free-market being ‘unable to promote social justice’ and to desiring ‘minimal Governmental interference in their citizen’s lives’.


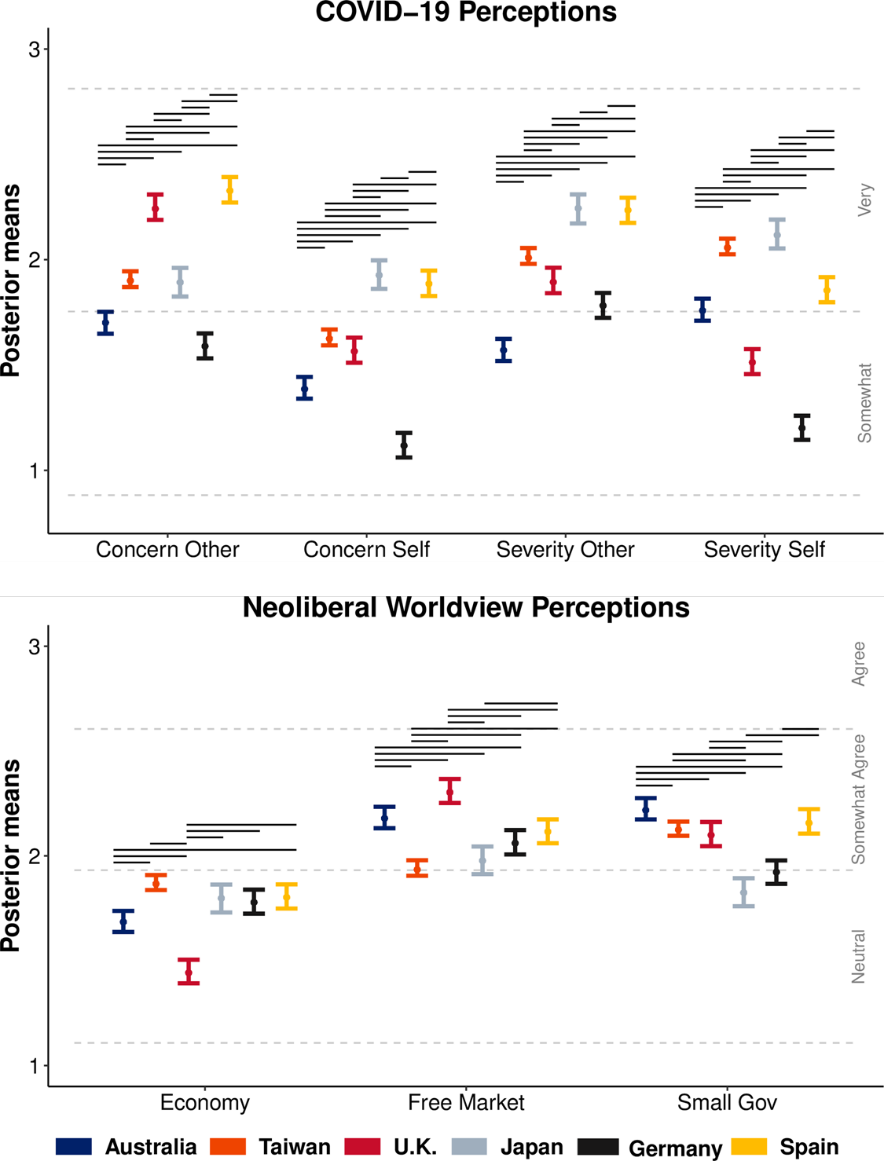


*Figure B.1.* Ordinal regression mean posterior distributions and latent Likert-ratings for COVID-19 perceptions (top) and world view perceptions across countries (bottom). Error bars display the 95% highest posterior density interval.

## Alternative models

Alternative models were fit to the immunity passport support items including an ordinal probit model assessing immunity passport support post immunity passport questions. This probit model used the original ordinal categories, with each intercept representing a boundary between Likert categories. For example, intercept 1 would represents the lowest boundary between ’none’ and ’a little’, while intercept 5 would represent the boundary between ’a lot’ and ’extremely’. Additionally, we modeled a binary and ordinal account of immunity passport support as measured prior to answering the immunity passport questions - the reader can think of this as participant’s ‘gut reaction’, while the findings presented in the main paper reflect attitudes after having thought about the risks and benefits that accompany immunity passports. The following details the results of these alternative modelling accounts. Each model was instantiated with four chains, 2000 iterations each with 1000 burn-ins each, using non-informative priors for the intercept, with fixed effects estimated from weakly informative Laplacian distributed priors centered on 0 with a scale parameter of 1. For ease of reading, bold parameters denote where the 95% credible intervals do not cross zero, thus indicating an effect. Odds ratios were not calculated for the probit models; doing so would be a misrepresentation of the data as the probit account does not measure the log odds of moving between response categories, however, this would be true for an ordinal logistic regression.

### Ordinal probit model of support measured after the immunity passport questions.

Table B.1 *Ordinal probit model of immunity passport acceptance after answering immunity passport items.*

| **Parameter** | **Estimate** | **Error** | **Lower 95% CI** | **Upper 95% CI** |
| --- | --- | --- | --- | --- |
| Intercept[1] | -1.11 | 0.41 | -2.01 | -0.25 |
| Intercept[2] | -0.16 | 0.41 | -1.06 | 0.7 |
| Intercept[3] | 0.68 | 0.41 | -0.22 | 1.54 |
| Intercept[4] | 1.83 | 0.41 | 0.94 | 2.69 |
| Intercept[5] | 2.91 | 0.41 | 2.02 | 3.76 |
| Age | -0.01 | 0.01 | -0.03 | 0.01 |
| GenderWoman | -0.02 | 0.02 | -0.06 | 0.02 |
| EducationHighschoolorGreater | -0.01 | 0.04 | -0.08 | 0.07 |
| **EducationUniversityorEquivalent** | -0.09 | 0.04 | -0.16 | -0.01 |
| **COVID_PositiveTRUE** | 0.24 | 0.07 | 0.1 | 0.37 |
| COVID_PositiveOtherTRUE | 0.02 | 0.03 | -0.04 | 0.08 |
| CumDeaths | 0.02 | 0.02 | -0.02 | 0.07 |
| CumCases | -0.01 | 0.02 | -0.05 | 0.03 |
| TrackingTechnologyTRUE | 0.27 | 0.43 | -0.67 | 1.14 |
| MaskUsageTRUE | -0.25 | 0.35 | -0.96 | 0.51 |
| LockdownTRUE | 0.11 | 0.34 | -0.63 | 0.8 |
| GovEffectiveness | -0.01 | 0.18 | -0.37 | 0.41 |
| IndexOfCommunality | -0.26 | 0.24 | -0.74 | 0.25 |
| **COVID_SeveritySelf** | 0.04 | 0.01 | 0.02 | 0.06 |
| COVID_SeverityOther | 0 | 0.01 | -0.02 | 0.02 |
| COVID_ConcernSelf | 0.02 | 0.02 | 0 | 0.06 |
| **COVID_ConcernOther** | 0.04 | 0.01 | 0.01 | 0.07 |
| **WVeconomy** | 0.08 | 0.01 | 0.07 | 0.1 |
| **WVfreemarket** | 0.07 | 0.01 | 0.06 | 0.09 |
| **WVsmallgov** | -0.04 | 0.01 | -0.05 | -0.02 |
| **IP_Concerned** | -0.28 | 0.01 | -0.31 | -0.25 |
| **IP_Like** | 0.61 | 0.01 | 0.59 | 0.64 |
| **IP_HarmSociety** | -0.25 | 0.01 | -0.27 | -0.22 |
| **IP_Fair** | 0.53 | 0.01 | 0.5 | 0.55 |
| **IP_InfectSelf** | 0.23 | 0.01 | 0.21 | 0.25 |

### Binomial model of support before immunity passport questions

Table B.2

*Binomial model of immunity passport acceptance before answering immunity passport items. Odds ratio denotes the multiplicative increase each coefficient confers to immunity passport support, calculated as the natural exponent of the estimate.*

| **Parameter** | **Estimate** | **Odds Ratio** | **Error** | **Lower 95% CI** | **Upper 95% CI** |
| --- | --- | --- | --- | --- | --- |
| Intercept | -0.06 | 0.94 | 0.64 | -1.63 | 0.99 |
| **Age** | 0.12 | 1.13 | 0.03 | 0.07 | 0.18 |
| GenderWoman | 0.02 | 1.02 | 0.05 | -0.08 | 0.11 |
| EducationHighschoolorGreater | 0.11 | 1.12 | 0.1 | -0.08 | 0.31 |
| EducationUniversityorEquivalent | 0.15 | 1.16 | 0.1 | -0.03 | 0.35 |
| COVID_PositiveTRUE | -0.26 | 0.77 | 0.17 | -0.59 | 0.06 |
| COVID_PositiveOtherTRUE | 0.11 | 1.12 | 0.08 | -0.05 | 0.28 |
| CumDeaths | -0.02 | 0.98 | 0.05 | -0.13 | 0.08 |
| **CumCases** | 0.07 | 1.07 | 0.05 | -0.03 | 0.18 |
| **TrackingTechnologyTRUE** | 0.09 | 1.09 | 0.59 | -1.25 | 1.26 |
| MaskUsageTRUE | -0.5 | 0.61 | 0.45 | -1.37 | 0.52 |
| LockdownTRUE | -0.16 | 0.85 | 0.47 | -1.13 | 0.91 |
| GovEffectiveness | 0.21 | 1.23 | 0.26 | -0.4 | 0.72 |
| IndexOfCommunality | -0.69 | 0.5 | 0.43 | -1.38 | 0.33 |
| **COVID_SeveritySelf** | 0.08 | 1.08 | 0.03 | 0.01 | 0.14 |
| COVID_SeverityOther | 0.06 | 1.06 | 0.03 | 0 | 0.12 |
| COVID_ConcernSelf | -0.05 | 0.95 | 0.04 | -0.13 | 0.02 |
| **COVID_ConcernOther** | 0.15 | 1.16 | 0.04 | 0.08 | 0.22 |
| **WVeconomy** | 0.14 | 1.15 | 0.02 | 0.11 | 0.18 |
| **WVfreemarket** | 0.15 | 1.16 | 0.02 | 0.12 | 0.19 |
| WVsmallgov | -0.03 | 0.97 | 0.02 | -0.07 | 0 |
| **IP_Concerned** | -0.63 | 0.53 | 0.04 | -0.7 | -0.56 |
| **IP_Like** | 1.19 | 3.29 | 0.03 | 1.13 | 1.26 |
| **IP_HarmSociety** | -0.32 | 0.73 | 0.03 | -0.38 | -0.25 |
| **IP_Fair** | 0.57 | 1.77 | 0.03 | 0.5 | 0.63 |
| **IP_InfectSelf** | -0.02 | 0.98 | 0.03 | -0.08 | 0.04 |

### Ordinal probit model of support before immunity passport questions

Table B.3

| **Parameter** | **Estimate** | **Error** | **Lower 95% CI** | **Upper 95% CI** |
| --- | --- | --- | --- | --- |
| Intercept[1] | -1.66 | 0.45 | -2.49 | -0.47 |
| Intercept[2] | -0.9 | 0.45 | -1.73 | 0.29 |
| Intercept[3] | -0.09 | 0.45 | -0.92 | 1.09 |
| Intercept[4] | 1.08 | 0.45 | 0.25 | 2.27 |
| Intercept[5] | 2.18 | 0.45 | 1.34 | 3.36 |
| **Age** | 0.04 | 0.01 | 0.02 | 0.06 |
| Gender (Woman) | 0.03 | 0.02 | -0.01 | 0.07 |
| Education: High School | -0.03 | 0.04 | -0.1 | 0.05 |
| **Education: University** | 0 | 0.04 | -0.08 | 0.07 |
| **COVID Positive Self** | -0.08 | 0.07 | -0.21 | 0.06 |
| **COVID Positive Other** | 0.08 | 0.03 | 0.02 | 0.14 |
| COVID Deaths (cumulative) | 0.02 | 0.02 | -0.02 | 0.06 |
| COVID Cases (cumulative) | 0 | 0.02 | -0.04 | 0.04 |
| Tracking Technology in Use | 0.05 | 0.52 | -1.06 | 1.11 |
| Masks in Use | -0.29 | 0.37 | -0.98 | 0.53 |
| Lockdown in Use | -0.06 | 0.37 | -0.82 | 0.82 |
| Gov Effectiveness | 0.06 | 0.2 | -0.42 | 0.47 |
| Index Of Communality | -0.33 | 0.27 | -0.81 | 0.39 |
| **COVID Severity Self** | 0.05 | 0.01 | 0.02 | 0.07 |
| **COVID Severity Other** | 0.04 | 0.01 | 0.01 | 0.06 |
| COVID Concern Self | -0.02 | 0.02 | -0.05 | 0 |
| **COVID Concern Other** | 0.08 | 0.01 | 0.05 | 0.1 |
| **Neoliberal WV: Economy** | 0.07 | 0.01 | 0.06 | 0.09 |
| **Neoliberal WV: Freemarket** | 0.08 | 0.01 | 0.07 | 0.09 |
| **Neoliberal WV: Small Gov** | -0.02 | 0.01 | -0.03 | 0 |
| **I.P Concerned** | -0.38 | 0.01 | -0.41 | -0.36 |
| **I.P Like** | 0.69 | 0.01 | 0.67 | 0.72 |
| **I.P Harm Society** | -0.17 | 0.01 | -0.19 | -0.14 |
| **I.P Fair** | 0.29 | 0.01 | 0.26 | 0.31 |
| **I.P InfectSelf** | -0.02 | 0.01 | -0.05 | 0 |

*Ordinal probit model of immunity passport acceptance before answering immunity passport items.*
