# Supplementary material for: Papers Please - Predictive Factors of National and International Attitudes Toward Immunity and Vaccination Passports: Online Representative Surveys
Source: JMIR Public Health Surveill. 2022 Jul 15;8(7):e32969. doi: 10.2196/32969 (PMC9290331; doi:10.2196/32969)
Supplement: Multimedia Appendix 3 [file publichealth_v8i7e32969_app3.docx]

# Multimedia Appendix 3. Model Results

## Binomial model parameters (main model reported in text)

Table C.1

*Parameter coefficients for the binomial predictive model presented in text. All Rhat values were 1. * Denotes random intercepts. Odds ratio denotes the multiplicative increase each coefficient confers to immunity passport support, calculated as the natural exponent of the estimate.*

| **Paramater** | **Estimate** | **Odds Ratio** | **Error** | **Lower 95% CI** | **Upper 95% CI** |
| --- | --- | --- | --- | --- | --- |
| Global Intercept | -1.67 | 0.19 | 0.69 | -3.14 | -0.32 |
| Japan* | -0.66 | 0.52 | 0.67 | -2.10 | 0.57 |
| Spain* | -0.20 | 0.82 | 0.86 | -2.00 | 1.54 |
| Australia* | -0.07 | 0.93 | 0.67 | -1.40 | 1.33 |
| United Kingdom* | 0.07 | 1.08 | 0.73 | -1.49 | 1.56 |
| Germany* | 0.11 | 1.11 | 0.66 | -1.29 | 1.38 |
| Taiwan* | 0.61 | 1.84 | 0.78 | -0.79 | 2.31 |
| Age | 0.03 | 1.03 | 0.03 | -0.02 | 0.08 |
| Gender (Woman) | -0.11 | 0.9 | 0.05 | -0.20 | -0.02 |
| Education: High School | 0.14 | 1.15 | 0.10 | -0.05 | 0.32 |
| Education: University | -0.03 | 0.97 | 0.09 | -0.21 | 0.15 |
| COVID Positive Self | 0.21 | 1.23 | 0.17 | -0.11 | 0.55 |
| COVID Positive Other | -0.01 | 0.99 | 0.08 | -0.16 | 0.14 |
| COVID Deaths (cumulative) | -0.03 | 0.97 | 0.05 | -0.13 | 0.08 |
| COVID Cases (cumulative) | 0.06 | 1.06 | 0.05 | -0.04 | 0.17 |
| Tracking Technology in Use | 0.58 | 1.79 | 0.70 | -0.78 | 1.90 |
| Masks in Use | -0.47 | 0.63 | 0.56 | -1.55 | 0.63 |
| Lockdowns in Use | 0.26 | 1.30 | 0.54 | -0.85 | 1.31 |
| Government Effectivness | 0.00 | 1.00 | 0.29 | -0.58 | 0.61 |
| Index of Individuality | -0.42 | 0.66 | 0.42 | -1.23 | 0.45 |
| COVID Severity Self | 0.07 | 1.07 | 0.03 | 0.01 | 0.13 |
| COVID Severity Other | 0.00 | 1.00 | 0.03 | -0.06 | 0.05 |
| COVID Concern Self | 0.07 | 1.07 | 0.04 | 0.00 | 0.15 |
| COVID Concern Other | 0.05 | 1.05 | 0.04 | -0.01 | 0.12 |
| Neoliberal WV: Economy | 0.16 | 1.17 | 0.02 | 0.13 | 0.20 |
| Neoliberal WV: Free Market | 0.16 | 1.17 | 0.02 | 0.12 | 0.19 |
| Neoliberal WV: Small Gov | -0.05 | 0.95 | 0.02 | -0.09 | -0.02 |
| I.P Concern | -0.50 | 0.61 | 0.03 | -0.56 | -0.43 |
| I.P Like | 1.02 | 2.77 | 0.03 | 0.96 | 1.08 |
| I.P Harm | -0.34 | 0.71 | 0.03 | -0.40 | -0.28 |
| I.P Fair | 0.92 | 2.51 | 0.03 | 0.86 | 0.98 |
| I.P Infect Self | 0.47 | 1.60 | 0.03 | 0.41 | 0.52 |

## Models by country

The following details the results of Bayesian generalized linear models completed separately for each country. Parameters that did not vary within a country (e.g., Government effectiveness was a point-estimate measure that varied between but not within countries), were excluded from the models. Each model was instantiated with four chains, 2000 iterations each with 1000 burn-ins each, using non-informative priors for the intercept, with fixed effects estimated from weakly informative Laplacian distributed priors centered on 0 with a scale parameter of 1. The following tables present estimates for each country, separately. For ease of reading, bold parameters denote where the 95% credible intervals do not cross zero, indicating an effect.

Table C.2

*Parameter coefficients for the binomial predictive model of immunity passport support as assessed for Australia. All Rhat values were 1. Odds ratio denotes the multiplicative increase each coefficient confers to immunity passport support, calculated as the natural exponent of the estimate.*

| **Parameter** | **Estimate** | **Odds ratio** | **Error** | **Lower 95% CI** | **Upper 95% CI** |
| --- | --- | --- | --- | --- | --- |
| Intercept | -1.16 | 0.31 | 1.17 | -3.38 | 1.29 |
| Age | 0.08 | 1.08 | 0.07 | -0.07 | 0.23 |
| Gender (Woman) | -0.24 | 0.79 | 0.14 | -0.51 | 0.03 |
| Education: High School | 0.05 | 1.05 | 0.2 | -0.34 | 0.45 |
| Education: University | -0.08 | 0.92 | 0.2 | -0.48 | 0.29 |
| COVID Positive Self | 0.84 | 2.32 | 0.46 | -0.04 | 1.78 |
| COVID Positive Other | 0.27 | 1.31 | 0.22 | -0.14 | 0.71 |
| COVID Deaths (cumulative) | -0.03 | 0.97 | 0.83 | -1.69 | 1.71 |
| COVID Cases (cumulative) | -0.47 | 0.63 | 0.63 | -1.85 | 0.67 |
| Tracking Technology in Use | 0.32 | 1.38 | 1.12 | -1.86 | 2.88 |
| Masks in Use | 0.34 | 1.4 | 1.2 | -2.04 | 3.05 |
| Lockdown in Use | -0.36 | 0.7 | 1.16 | -2.93 | 1.81 |
| COVID Severity Self | 0.09 | 1.09 | 0.09 | -0.07 | 0.27 |
| COVID Severity Other | 0.04 | 1.04 | 0.08 | -0.12 | 0.2 |
| COVID Concern Self | 0.21 | 1.23 | 0.12 | -0.02 | 0.44 |
| COVID Concern Other | -0.07 | 0.93 | 0.11 | -0.28 | 0.14 |
| **Neoliberal WV: Economy** | 0.27 | 1.31 | 0.06 | 0.16 | 0.38 |
| Neoliberal WV: Freemarket | 0.08 | 1.08 | 0.06 | -0.05 | 0.21 |
| **Neoliberal WV: Small Gov** | -0.13 | 0.88 | 0.05 | -0.23 | -0.02 |
| **I.P Concerned** | -0.45 | 0.64 | 0.1 | -0.63 | -0.27 |
| **I.P Like** | 1.31 | 3.71 | 0.09 | 1.14 | 1.48 |
| I.P Harm Society | -0.16 | 0.85 | 0.09 | -0.34 | 0.01 |
| **I.P Fair** | 1.23 | 3.42 | 0.09 | 1.05 | 1.41 |
| **I.P InfectSelf** | 0.71 | 2.03 | 0.08 | 0.55 | 0.87 |

Table C.3

*Parameter coefficients for the binomial predictive model of immunity passport support as assessed for Germany. All Rhat values were 1. Odds ratio denotes the multiplicative increase each coefficient confers to immunity passport support, calculated as the natural exponent of the estimate.*

| **Parameter** | **Estimate** | **Odds ratio** | **Est.Error** | **Lower 95% CII** | **Upper 95% CI** |
| --- | --- | --- | --- | --- | --- |
| Intercept | -1.03 | 0.36 | 0.39 | -1.8 | -0.27 |
| Age | 0.09 | 1.09 | 0.08 | -0.07 | 0.26 |
| Gender (Woman) | -0.09 | 0.91 | 0.13 | -0.35 | 0.17 |
| Education: High School | -0.01 | 0.99 | 0.17 | -0.35 | 0.33 |
| Education: University | 0.2 | 1.22 | 0.21 | -0.19 | 0.63 |
| COVID Positive Self | 0.17 | 1.19 | 0.32 | -0.45 | 0.81 |
| COVID Positive Other | -0.18 | 0.84 | 0.17 | -0.52 | 0.14 |
| COVID Deaths (cumulative) | 0.36 | 1.43 | 0.49 | -0.48 | 1.45 |
| COVID Cases (cumulative) | -0.43 | 0.65 | 0.49 | -1.54 | 0.42 |
| COVID Severity Self | 0.1 | 1.11 | 0.1 | -0.09 | 0.29 |
| COVID Severity Other | -0.16 | 0.85 | 0.09 | -0.34 | 0.01 |
| COVID Concern Self | -0.06 | 0.94 | 0.11 | -0.29 | 0.16 |
| COVID Concern Other | 0.12 | 1.13 | 0.1 | -0.07 | 0.32 |
| **Neoliberal WV: Economy** | 0.21 | 1.23 | 0.05 | 0.11 | 0.31 |
| **Neoliberal WV: Freemarket** | 0.13 | 1.14 | 0.06 | 0.02 | 0.24 |
| **Neoliberal WV: Small Gov** | -0.11 | 0.9 | 0.05 | -0.21 | -0.01 |
| **I.P Concerned** | -0.28 | 0.76 | 0.1 | -0.48 | -0.09 |
| **I.P Like** | 1.3 | 3.67 | 0.1 | 1.11 | 1.5 |
| **I.P Harm Society** | -0.32 | 0.73 | 0.1 | -0.5 | -0.13 |
| **I.P Fair** | 0.86 | 2.36 | 0.09 | 0.69 | 1.03 |
| **I.P InfectSelf** | 0.46 | 1.58 | 0.09 | 0.29 | 0.63 |

Table C.4

*Parameter coefficients for the binomial predictive model of immunity passport support as assessed for Japan. All Rhat values were 1. Odds ratio denotes the multiplicative increase each coefficient confers to immunity passport support, calculated as the natural exponent of the estimate.*

| **Parameter** | **Estimate** | **Odds ratio** | **Est.Error** | **Lower 95% CII** | **Upper 95% CI** |
| --- | --- | --- | --- | --- | --- |
| Intercept | -2.45 | 0.09 | 0.73 | -3.91 | -1 |
| Age | 0.06 | 1.06 | 0.09 | -0.12 | 0.25 |
| Gender (Woman) | 0.01 | 1.01 | 0.17 | -0.31 | 0.34 |
| Education: High School | -0.46 | 0.63 | 0.43 | -1.36 | 0.29 |
| Education: University | -0.62 | 0.54 | 0.43 | -1.5 | 0.13 |
| COVID Positive Self | 1.09 | 2.97 | 0.88 | -0.4 | 2.98 |
| COVID Positive Other | -1.47 | 0.23 | 0.97 | -3.62 | 0.09 |
| COVID Deaths (cumulative) | -0.06 | 0.94 | 0.7 | -1.52 | 1.4 |
| COVID Cases (cumulative) | -0.05 | 0.95 | 0.7 | -1.49 | 1.42 |
| COVID Severity Self | 0.17 | 1.19 | 0.11 | -0.04 | 0.39 |
| COVID Severity Other | -0.06 | 0.94 | 0.11 | -0.27 | 0.15 |
| COVID Concern Self | -0.23 | 0.79 | 0.15 | -0.53 | 0.06 |
| **COVID Concern Other** | 0.39 | 1.48 | 0.16 | 0.08 | 0.71 |
| Neoliberal WV: Economy | 0.01 | 1.01 | 0.1 | -0.17 | 0.2 |
| **Neoliberal WV: Freemarket** | 0.22 | 1.25 | 0.1 | 0.01 | 0.41 |
| Neoliberal WV: Small Gov | 0.01 | 1.01 | 0.08 | -0.16 | 0.18 |
| **I.P Concerned** | -0.46 | 0.63 | 0.13 | -0.72 | -0.2 |
| **I.P Like** | 0.94 | 2.56 | 0.11 | 0.72 | 1.16 |
| I.P Harm Society | -0.09 | 0.91 | 0.11 | -0.31 | 0.13 |
| **I.P Fair** | 0.6 | 1.82 | 0.11 | 0.39 | 0.81 |
| **I.P InfectSelf** | 0.7 | 2.01 | 0.1 | 0.52 | 0.89 |

Table C.5

*Parameter coefficients for the binomial predictive model of immunity passport support as assessed for Spain. All Rhat values were 1. Odds ratio denotes the multiplicative increase each coefficient confers to immunity passport support, calculated as the natural exponent of the estimate.*

| **Parameter** | **Estimate** | **Odds ratio** | **Est.Error** | **Lower 95% CII** | **Upper 95% CI** |
| --- | --- | --- | --- | --- | --- |
| Intercept | -1.45 | 0.23 | 0.42 | -2.27 | -0.63 |
| Age | 0.03 | 1.03 | 0.08 | -0.13 | 0.19 |
| Gender (Woman) | 0.05 | 1.05 | 0.14 | -0.22 | 0.32 |
| Education: High School | 0.39 | 1.48 | 0.23 | -0.03 | 0.84 |
| Education: University | 0.06 | 1.06 | 0.22 | -0.36 | 0.51 |
| COVID Positive Self | -0.11 | 0.9 | 0.29 | -0.69 | 0.48 |
| COVID Positive Other | -0.01 | 0.99 | 0.14 | -0.29 | 0.27 |
| COVID Deaths (cumulative) | 0.16 | 1.17 | 0.23 | -0.26 | 0.64 |
| COVID Cases (cumulative) | -0.16 | 0.85 | 0.23 | -0.64 | 0.26 |
| COVID Severity Self | -0.08 | 0.92 | 0.1 | -0.27 | 0.11 |
| COVID Severity Other | 0.1 | 1.11 | 0.09 | -0.07 | 0.29 |
| COVID Concern Self | 0.04 | 1.04 | 0.12 | -0.19 | 0.27 |
| COVID Concern Other | -0.02 | 0.98 | 0.1 | -0.22 | 0.18 |
| **Neoliberal WV: Economy** | 0.11 | 1.12 | 0.05 | 0.01 | 0.21 |
| **Neoliberal WV: Freemarket** | 0.25 | 1.28 | 0.05 | 0.14 | 0.35 |
| **Neoliberal WV: Small Gov** | -0.16 | 0.85 | 0.06 | -0.27 | -0.05 |
| **I.P Concerned** | -0.61 | 0.54 | 0.11 | -0.82 | -0.4 |
| **I.P Like** | 1.19 | 3.29 | 0.1 | 1.01 | 1.38 |
| I.P Harm Society | -0.1 | 0.9 | 0.1 | -0.31 | 0.1 |
| **I.P Fair** | 1.16 | 3.19 | 0.09 | 0.99 | 1.35 |
| **I.P InfectSelf** | 0.64 | 1.9 | 0.09 | 0.46 | 0.83 |

Table C.6

*Parameter coefficients for the binomial predictive model of immunity passport support as assessed for Taiwan. All Rhat values were 1. Odds ratio denotes the multiplicative increase each coefficient confers to immunity passport support, calculated as the natural exponent of the estimate.*

| **Parameter** | **Estimate** | **Odds ratio** | **Est.Error** | **Lower 95% CII** | **Upper 95% CII** |
| --- | --- | --- | --- | --- | --- |
| Intercept | -1.62 | 0.2 | 0.31 | -2.27 | -1.04 |
| Age | 0.06 | 1.06 | 0.04 | -0.01 | 0.13 |
| Gender (Woman) | -0.09 | 0.91 | 0.07 | -0.23 | 0.05 |
| Education: High School | 0.41 | 1.51 | 0.28 | -0.1 | 1 |
| Education: University | 0.06 | 1.06 | 0.26 | -0.42 | 0.62 |
| COVID Positive Self | -0.29 | 0.75 | 0.42 | -1.13 | 0.47 |
| COVID Positive Other | -0.11 | 0.9 | 0.2 | -0.53 | 0.28 |
| COVID Deaths (cumulative) | -0.01 | 0.99 | 0.06 | -0.12 | 0.09 |
| COVID Cases (cumulative) | 0.1 | 1.11 | 0.06 | 0 | 0.21 |
| COVID Severity Self | 0.05 | 1.05 | 0.04 | -0.03 | 0.14 |
| COVID Severity Other | 0.01 | 1.01 | 0.04 | -0.08 | 0.09 |
| **COVID Concern Self** | 0.13 | 1.14 | 0.05 | 0.03 | 0.24 |
| COVID Concern Other | 0.03 | 1.03 | 0.05 | -0.07 | 0.13 |
| **Neoliberal WV: Economy** | 0.16 | 1.17 | 0.03 | 0.11 | 0.22 |
| **Neoliberal WV: Freemarket** | 0.15 | 1.16 | 0.03 | 0.09 | 0.21 |
| Neoliberal WV: Small Gov | 0 | 1 | 0.03 | -0.06 | 0.06 |
| **I.P Concerned** | -0.5 | 0.61 | 0.04 | -0.59 | -0.42 |
| **I.P Like** | 0.72 | 2.05 | 0.04 | 0.64 | 0.8 |
| **I.P Harm Society** | -0.46 | 0.63 | 0.04 | -0.54 | -0.37 |
| **I.P Fair** | 0.82 | 2.27 | 0.04 | 0.74 | 0.9 |
| **I.P InfectSelf** | 0.32 | 1.38 | 0.04 | 0.24 | 0.39 |

Table C.7

*Parameter coefficients for the binomial predictive model of immunity passport support as assessed for the United Kingdom. All Rhat values were 1. Odds ratio denotes the multiplicative increase each coefficient confers to immunity passport support, calculated as the natural exponent of the estimate.*

| **Parameter** | **Estimate** | **Odds ratio** | **Est.Error** | **Lower 95% CII** | **Upper 95% CI** |
| --- | --- | --- | --- | --- | --- |
| Intercept | -0.6 | 0.55 | 0.6 | -1.8 | 0.58 |
| Age | 0.07 | 1.07 | 0.09 | -0.12 | 0.26 |
| Gender (Woman) | -0.2 | 0.82 | 0.17 | -0.51 | 0.12 |
| Education: High School | -0.26 | 0.77 | 0.26 | -0.81 | 0.23 |
| Education: University | -0.06 | 0.94 | 0.22 | -0.5 | 0.36 |
| COVID Positive Self | 0.36 | 1.43 | 1.22 | -1.75 | 3.27 |
| COVID Positive Other | 0.19 | 1.21 | 0.2 | -0.18 | 0.6 |
| COVID Deaths (cumulative) | -0.74 | 0.48 | 1.19 | -3.76 | 1.13 |
| COVID Cases (cumulative) | -0.71 | 0.49 | 1.13 | -3.37 | 1.2 |
| COVID Severity Self | 0.06 | 1.06 | 0.11 | -0.15 | 0.27 |
| COVID Severity Other | -0.03 | 0.97 | 0.09 | -0.22 | 0.15 |
| COVID Concern Self | 0.05 | 1.05 | 0.12 | -0.19 | 0.29 |
| COVID Concern Other | 0.07 | 1.07 | 0.11 | -0.15 | 0.3 |
| Neoliberal WV: Economy | 0.1 | 1.11 | 0.06 | -0.03 | 0.22 |
| Neoliberal WV: Freemarket | 0.06 | 1.06 | 0.08 | -0.09 | 0.22 |
| Neoliberal WV: Small Gov | -0.03 | 0.97 | 0.06 | -0.16 | 0.09 |
| **I.P Concerned** | -0.57 | 0.57 | 0.12 | -0.81 | -0.33 |
| **I.P Like** | 1.7 | 5.47 | 0.12 | 1.46 | 1.95 |
| **I.P Harm Society** | -0.64 | 0.53 | 0.11 | -0.87 | -0.43 |
| **I.P Fair** | 1.15 | 3.16 | 0.12 | 0.92 | 1.39 |
| I.P InfectSelf | 0.13 | 1.14 | 0.09 | -0.03 | 0.31 |
